# M2c Macrophages enhance phalange regeneration of amputated mice digits in an organ co-culture system

**DOI:** 10.22038/IJBMS.2021.57887.12870

**Published:** 2021-11

**Authors:** Fatemeh Bijarchian, Leila Taghiyar, Zahra Azhdari, Mohamadreza Baghaban Eslaminejad

**Affiliations:** 1 Department of Stem Cells and Developmental Biology, Cell Science Research Center, Royan Institute for Stem Cell Biology and Technology, ACECR, Tehran, 1665659911, Iran; 2 Department of Developmental Biology, University of Science and Culture, Tehran, Iran

**Keywords:** Anti-inflammatory – responses, IL-10, M2c macrophage, Organ-culture, Paw regeneration, TGF-β

## Abstract

**Objective(s):**

Delayed anti-inflammatory responses and scar-formation are the main causes for inability of injured body parts such as phalanges to regrow in mammals. Salamanders can regenerate fully scar-free body structures, followed by the appearance of anti-inflammatory responses at the injured site immediately after amputation. This study aimed to evaluate the local regenerative effects of direct amplified anti-inflammatory signals on regeneration of amputated mice digit tips using M2c-macrophages in a co-cultured organ system for the first time.

**Materials and Methods:**

We used the amputated digits from the paws of 18.5E day old C57BL/6J mice. Monocytes were obtained from peripheral blood and co-cultured with amputated digits, which subsequently enhanced the M2c macrophage phenotype induced by IL-10. We also examined the regenerative effects of IL-10 and transcription growth factor-beta 1 (TGF-β1).

**Results:**

The regrowth of new tissue occurred 10 days post-amputation in all groups. This regrowth was related to enhanced Msh homeobox-1 (*Msx1*), Msh homeobox-2 (*Msx2*), and bone morphogenic protein-4 (*Bmp4*) genes. Increased expression of fibroblast growth factor-8 (*Fgf-8*) also increased the proliferation rate. Histological analyses indicated that epidermal-closure occurred at 3-dpa in all groups. We observed full digit tip regeneration in the co-cultured group. Particularly, there was new tissue regrowth observed with 40 µg/ml of IL-10 and 120 µg/ml of TGF-β. In contrast, the control group had no remarkable digit elongation.

**Conclusion:**

We propose that a direct amplified anti-inflammatory response at the digit injury site can regenerate epithelial and mesenchymal tissues, and might be useful for limb regeneration without scar formation in adult mammals.

## Introduction

Limb regeneration in mammals is followed by tissue fibrosis or scar formation due to instant, robust promotion of pro-inflammatory responses or delayed amplification of anti-inflammatory signals after amputation (1). Currently, neutrophils and macrophages are the key cell-derived mediators of the inflammatory process. In addition, immunological signaling is defined as the essential molecules needed for full limb restoration in adult salamanders, which have the capability to regenerate complex structures such as the limbs, lens, heart, and tails (2, 3). In particular, injured salamanders undergo limb regeneration via a remarkable scar-free repair process that occurs in response to amputation anywhere along the limb axis (4). Indeed, both limb development and regeneration are applied by up-regulation of the same signaling pathway in both amphibians and mammalians (5). For example, the transforming growth factor-beta (TGF-β) signaling pathway plays the main role in wound healing processes and immune response. During normal limb development, bone morphogenetic protein (BMP) signaling pathways are required for apical ectodermal ridge (AER) formation, a multilayer epithelium that is necessary for mesenchymal cell proliferation under that, as well as osteogenesis, control of cell death, and proliferation (6, 7). In addition, the proximo-distal limb patterning following the ecto-mesodermal interaction is a key process of limb elongation which is controlled by FGF-8 expression in both limb regeneration and development. The Msx genes control the Fgf8 expression indirectly through BMP pathway stimulation (8). Msx genes such as Msx1 (HOX7) and Msx2 (HOX8) are the members of HOX family genes that show a key role in digit tip patterning and regeneration (9). On the other hand, inflammation and scar formation occur following limb amputation and blood clot formation events immediately after epithelium closure in mammalians. So, scar generation limits ecto-mesodermal interaction as well as essential signaling cascade for limb patterning and causes failure of limb regeneration in mammalians (10). Results of previous studies show that the time of immune cell infiltration is a critical factor in limb regrowth or fibrotic scar formation after an injury (11). Both leucocyte and lymphocyte subpopulations may attract keratinocytes and fibroblasts to activate pro-inflammatory responses during the initial 48–96 hr after an injury. Fibrotic scars mostly comprise types I and III collagen at the injured site and can cause an aberrant repair instead of full regeneration (12). Hence, early infiltrating immune cells play a crucial role during the first 24–96 hr after an injury and these cells determine the level of scar formation or whether full regeneration will occur. In contrast, initiation of scar-free wound healing, cell proliferation, angiogenesis, and subsequent full regeneration carried out bare the result of the influence of early anti-inflammatory signals that occur during the first 24 hr after amputation in salamanders. Extracellular matrix (ECM) remodeling, which is the main process in scar-free repair performed by myofibroblast activation, is the main ECM-producing cell in salamanders (13). 

Macrophages are the main immune cells that appear immediately after clot formation. Macrophages play numerous essential roles in wound healing such as resolution of inflammation, clearing the apoptotic cells, and the support of mesenchymal cell proliferation and tissue renewal following damage (14). Notably, the roles and necessity of macrophage infiltration and their cytokines have been established in full limb regeneration of salamanders (15). Nonetheless, this process is not understood in regeneration of mammalian limbs (16). Therefore, local amplification of anti-inflammatory macrophages or macrophage-derived therapeutic molecules such as interleukin 10 (IL-10) and transcription growth factor-beta 1 (TGF-β1) may perform a regeneration-permissive condition that promotes *de novo* regrowth of damaged body parts in mammals (17, 18). 

Subpopulations of M1 and M2 macrophages are the main sources of pro-inflammatory and anti-inflammatory signals, respectively (19, 20). M1 macrophages express pro-inflammatory molecules such as IL-23, IL-12, TNF-α, and major histocompatibility classes I and II (MHCI/II)(21). Conversely, a wide range of anti-inflammatory molecules, such as IL-10 and TGF-β1 are particularly released from M2c subtypes (22). In addition, IL-10 is a critical immune-regulatory mediator produced by a variety of cell types such as Th2, T-reg cells, and the anti-inflammatory M2c macrophages (23).

TGF-β is a multifunctional growth factor and a crucial regulator of embryonic development and wound healing, including activation of *Runx2* and fibronectin genes (24)(16). *Runx2* and fibronectin genes are mediators of matrix construction as well as bone formation during scar-free repair (25, 26). 

Interestingly, previous studies showed that induction of pro-inflammatory response using artificial stimulus caused scar formation in salamanders similar to mammals (27) (28). Macrophage-depleted axolotl limb stumps did not undergo full limb regeneration due to blockage of the TGF-β signaling pathway. Amplitude and maintenance of IL-10- and TGF-β-producing T-regs could suppress inflammatory responses in damaged tissues (29). 

In particular, recent evidence indicates that resident tissue immune cells in both human nail tissues and nail matrix are the site of immune privilege (IP) (30). IP is present in organs such as the eyes and the brain and protects them from excessive inflammation or activation of an autoimmune reaction as a result of tissue damage or pathogens (31-33). Regenerative limb potency is restricted to the distal tip of the phalanges in a region related to the nail organ in mammals (34). The tissue that surrounds the nail matrix shows prominent local expression of effective immunosuppressants such as TGF-β1 (35). Numerous pieces of evidence demonstrate that tissue-resident macrophage pools play the main role during tissue homeostasis in the heart, intestines, and kidneys (36-39). 

Immediately after amputation of the mouse digit tip, anti-inflammatory macrophages are needed to synchronize regeneration of this digit tip. In the laboratory, the whole organ culture system is an ideal model for limb/digit tip *ex vivo* studies. Novel culture methods which include 3D cultures, organoids, or organ chips (organs-on-chips) have better reproducibility and control of the tissue or organ microenvironment (40-43). More importantly, elimination of the innate immune responses leads to enhanced recognition of the M2c macrophage efficacy in organ culture assessment. 

Thus, the present study aimed to investigate the effect of macrophage in digit tip regeneration of mice *ex vivo*. 

**Figure 1:**
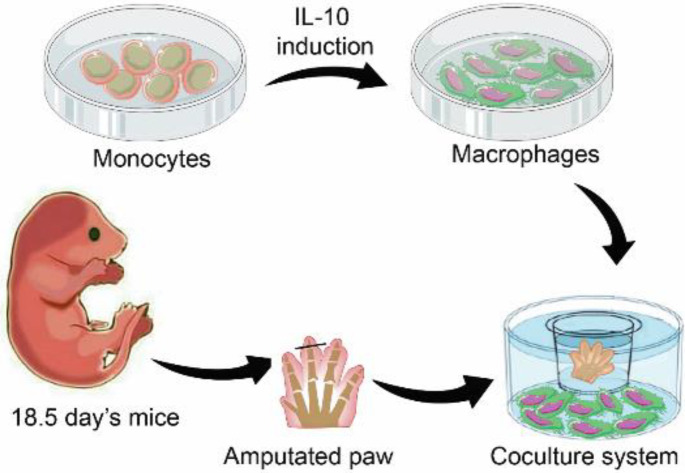
M2c-Macrophages co-cultured with mice amputated paw

Here, our designed co-cultured organ system consisted of the forelimbs of 18.5E day old C57BL/6J fetal mice and M2c macrophages. We used various concentrations of IL-10 and TGF-β1, as key anti-inflammatory cytokines, to assess their parallel regenerative effects on the amputated mouse digit tips. The M2c macrophages obtained from C57BL/6J peripheral blood monocytes were subsequently cultured, characterized, and expanded. Then, the amputated paws were cultured in 3 groups: co-culture with M2c macrophages (co-mac) and cultured using IL-10 (40, 80, and 120 ng/ml concentrations), and TGF-β1 (40, 80, and 120 ng/ml concentration (Figure 1). In this research, we used the fetal forelimb due to the lower thicknesses of the epidermal and dermal skin layers, which were appropriate for culture medium penetration. 

Our findings provided evidence for the functional roles of M2c macrophages in the regenerative process of an amputated digit tip. We found that 40 ng/ml and 120 ng/ml treatments were the best concentrations of IL-10 and TGF-β1, respectively. This experimental design enabled us to test the regenerative effects of local amplification of the M2c anti-inflammatory macrophage as well as IL-10 and TGF-β1 cytokines during the first 24 hr following limb amputation, when the ability to regenerate is generally lost. We observed improved regenerative capability of the phalanges along with the sustained presence of dose-dependent IL-10 and TGF-β1 cytokines and M2c macrophages in the amputated digits. However, the* ex vivo* method involves removing a pro-inflammatory response that blocked digit tip regeneration; but, the results suggested that we were able to improve the existence of anti-inflammation signals immediately after digit amputation.

## Materials and Methods

***Animals and organ harvest***

All procedures were approved by the Animal Ethics Committee at the animal department, of Royan Institute (Ethical license number: IR.ACECR.ROYAN.REC.1394.75) and were according to the international regulations of the European Directive 2010/63/EU and ethical guidelines for the study of experimental pain in conscious animals by the International Association of the Study of Pain. Approximately 6–8-week-old C57BL/6J male and female mice were used in this study. The animals were housed under stable laboratory conditions with a temperature of 22±5 °C and 50±5% humidity, a 12-hr day/night cycle, and free access to food and water. After mating, 18–18.5-day-old pregnant mice were anesthetized with 80 µl of ketamine and 120 µl of xylazine. All fetuses were collected and their forelimb wrists were identified and amputated under aseptic conditions. 

***Digit tip amputation and organ culture***

Digit tip amputation was performed on digits 2, 3, and 4 of the forelimbs. Approximately 1 mm at the proximal site of the digits were amputated to remove any generable levels. The amputated paws were placed on a 70 µm filter insert and subsequently transferred to 24-well tissue culture plates that contained Roswell Park Memorial Institute medium (RPMI 1640; Gibco) supplemented with 15% fetal bovine serum (FBS; Gibco; Germany), along with 10 000 units of penicillin and 10 mg/ml streptomycin (Sigma-Aldrich; Germany) to prevent bacterial contamination of the digit cultures. We divided the samples into three groups: amputated paws co-cultured with M2c macrophage (co-mac) and treatment groups that had three concentrations (40, 80, and 120 ng/ml) of IL-10 and TGF-β1. The extent of digit tip regeneration was measured on days 3 and 10 after cultivation.

***Peripheral blood mononuclear cell (PBMC) isolation and purification***

We isolated the monocular cells from peripheral blood according to the Sha *et al*. method (44). Briefly, after cardiac puncture in the anesthetized mice, we collected fresh whole peripheral blood in pre-filled heparin syringes. The blood samples were pooled in 15 ml centrifuge tubes. We isolated peripheral blood mononuclear cells (PBMC) from the blood by Ficoll® solution. Briefly, whole blood was diluted in phosphate-buffered saline (PBS; Gibco) and slowly loaded onto three ml of Ficoll-Paque Plus (Inno-train, Germany). RBCs were isolated by centrifugation at 1200 g for 40 min at 4 °C. The mononuclear cell phase that contained lymphocytes and monocytes was transferred to a sterile tube. The cells were centrifuged at 1200 g for 10 min and washed with PBS. 

***Monocyte differentiation, macrophage priming, and macrophage morphology***

We placed 2×10^6^ cells/ml of the purified cells into 48-well plates that contained 2–3 ml RPMI 1640 medium supplemented with 10% FBS. The cells were cultured with 20 ng/ml of IL-10, which enabled them to convert to M2c macrophages.

***M2c Macrophage characterization bacterial phagocytosis evaluation***

Bacterial phagocytosis activity was performed to confirm M2c cell generation. GFP-labeled *Escherichia* *coli* was freshly grown at 37 °C on agar plates supplemented with the appropriate antibiotics. After 24 hr, a single colony was grown in Lennox L broth-medium (Sigma-Aldrich) with shaking at 37 °C until it reached the mid-logarithmic phase (OD600 of ~0.4). The bacteria were washed with normal saline, suspended in PBS solution, and immediately placed onto the M2c macrophage cultured plate at 50:1 ratio of bacteria to macrophages for 40 min. The unattached free bacteria were washed, and the attached cells were fixed using 2% paraformaldehyde for 15–20 min. We used an inverted fluorescent microscope (Olympus BX51, Japan) to assess the bacteria that were phagocytosed by the M2c macrophages.

***Immunocytochemistry (ICC) and protein expression analysis***

Immunocytochemistry (ICC) was used to confirm the absence of CD86 and MHC I, the main markers of pro-inflammatory macrophages. Briefly, M2c macrophages were fixed in 4% paraformaldehyde (PFA) for 20 min. Next, 1% bovine serum albumin (BSA) in PBS was used to block the fixed cells for 30 min at room temperature (RT), then we added rat polyclonal anti-mouse CD86 (BD; Germany) and MHC II (eBioscience; Germany) primary antibodies, and the cells were incubated overnight at 4 °C. Subsequently, we added goat anti-rat Alexa Fluor® 488 secondary antibody (1:500, Invitrogen) for 60 min at RT. The cells were stained with DAPI nuclear counterstain (Invitrogen) and we observed the cells with a fluorescent microscope (Olympus BX51, Japan).

***Digit harvest and whole mount analysis***

The paws were harvested for assessment of growth ratios of the digit tips at 3 and 10 days post-amputation (dpa). The whole digit tip was used for the control group. In order to determine the mount digit elongation, a random number of digits was fixed overnight at 4 °C in a 10% formalin solution. After washing in 1% potassium hydroxide (KOH) in H_2_O, the digits were serially incubated in 20%–100% glycerol/1% KOH for 4–16 hr at RT. In order to measure new tissue regrowth, we measured the length of each distal phalanx (about 15 random digits for each group). 

***Histological analysis***

 Digits from the co-cultured experimental groups and IL-10 and TGF-β treatment samples were fixed in 10% formalin solution for 24 hr at 4 °C, then dehydrated in a series of ethanol solution. The paws were then paraffin-embedded and sliced into 6-μm thick sections for histological staining.

***Hematoxylin and eosin (H&E), masson’s trichrome, alizarin red s, and alcian blue staining***

At first, the specimens were deparaffinized in xylene, dehydrated through an ethanol series, and then stained with hematoxylin and eosin (H&E). We used Gill’s hematoxylin to stain the nuclei and acidified eosin to counterstain the cytoplasm according to standard procedures. Masson’s trichrome was used to selectively identify newly formed tissues (Gomori, procedure HT10, Sigma-Aldrich) according to the manufacturer’s instructions. In order to detect the bone and cartilage segments, we stained some sections with Alizarin red S for bone and Alcian blue for cartilage.

***Quantitative reverse transcription polymerase chain reaction (Qrt-PCR) measurements***

The expression levels of regenerative related genes Msh homeobox 1 (*Msx1)*, Msh homeobox 2 (*Msx2)*, fibroblast growth factor-8 (*Fgf-8)*, bone morphogenic protein 4 (*Bmp4),* and *Ki67* were evaluated by quantitative-reverse transcription-polymerase chain reaction (qRT-PCR). Total RNA was extracted from the cells by using the RNeasy Plus Universal Mini Kit (Qiagen, USA). cDNA was produced by the RevertAid First Strand cDNA Synthesis Kit (Fermentas, USA) according to the manufacturer’s instructions. qRT-PCR reactions were performed in duplicate using SensiMix™ SYBR® (Applied Biosystems Life Technologies, Inc., ref: 4367659) with the ABI StepOnePlus real-time PCR system (Applied Biosystems Life Technologies, Inc.) and analyzed with StepOne software (Applied Biosystems Life Technologies, version 2.1; USA). Three independent biological times were repeated for the specimens. The reference gene *GAPDH* was used to normalize the expression levels of the target genes. We used the comparative ΔΔCT method for data analysis. Table 1 lists the primers used in the experiments. 

*I* ***mmunohistochemistry (IHC) and protein expression analysis***

 Immunohistochemistry (IHC) was used to evaluate MSX1, MSX2, BMP4, and FGF8 protein expressions. Briefly, 10% BSA with 2% goat serum was used for 30 min to block any nonspecific antigens, followed by overnight incubation with primary antibodies MSX1 (Abcam), MSX2 (Abcam), BMP4 (eBioscience), and FGF8 (eBioscience) at 4 °C. The secondary antibody horseradish peroxidase (HRP; Invitrogen) was used at 1:5000 concentrations for 1 hr. The results were observed by a light microscope (Olympus, Japan).

***Statistical analysis***

Statistical analyses were performed on datasets of at least three independent experiments using an unpaired student’s t-test when comparing two groups. One-way ANOVA with Tukey’s multiple comparison test was used to compare more than two groups or two-way ANOVA with Tukey’s multiple comparison test for nonparametric results with GraphPad Prism software (GraphPad, San Diego, CA, USA). 

## Results

***Functionality assessments of macrophages induced to the m2c subtype***

**Figure 2.**
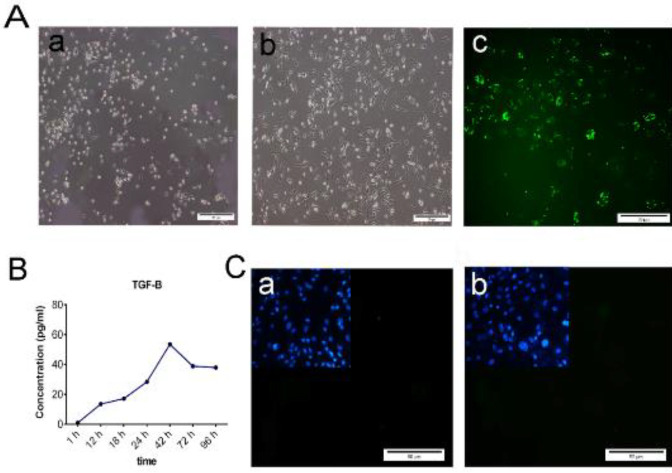
Functionality assessments of the M2c macrophage subtype. A. Mononuclear plastic-adherent cells were isolated from peripheral blood. Monocyte cells before interleukin 10 (IL-10) stimuli (a) and M2c subtype induced cells 10 days after IL-10 stimuli (b). The immunofluorescence image represents the green fluorescent protein (GFP+) bacterial phagocytosis (c). B. ELISA analysis shows the level of transcription growth factor-beta 1 (TGF-β1) secreted by M2c macrophages. C. Immunocytochemistry (ICC) images displayed the expression of CD86 and major histocompatibility class II (MHC II) as surface biomarkers (a, c). Scale bar: 50 μm

We considered the main properties of the M2c subtype of macrophages to establish immature monocytes that converted to M2c macrophages in response to IL-10 stimuli. We isolated mononuclear plastic-adherent cells from the peripheral blood of mice. The monocytes did not constitute a uniform population in the inactivated forms and showed a typical round shape (Figure 2Aa). The first morphological changes in the monocytes appeared within 3 to 5 days after *in vitro* stimulation by IL-10. The M2 macrophages are polarized cells with cytoplasmic appendages compared with monocytes (Figure 2Ab). Then, we evaluated the phagocytic activity in the *in vitro* culture to identify the M2c macrophage population. The *Ecoli*-green fluorescent protein (GFP)^+ ^bacteria phagocytosed by M2c macrophages in the culture plate were tracked using a fluorescent microscope (Olympus BX51, Japan; Figure 2Ac). In addition, we assessed for a specific marker that confirmed the isolated M2c subtype such as TGF-β1 as the main anti-inflammatory cytokine. ELISA analysis showed that TGF-β1 increased from 1 hr to 48 hr after IL-10 treatment (Figure 2B). The protein expression levels of the CD86 and MHC- II inflammatory markers of pro-macrophages were evaluated by immunofluorescence. The lack of expressions of the CD86 and MHC II markers confirmed the formation of the anti-inflammatory M2c macrophages (Figures 2Ca, b). 

***Development of an in vitro co-culture model of digit tip amputated forelimb with M2c macrophages***

**Figure 3:**
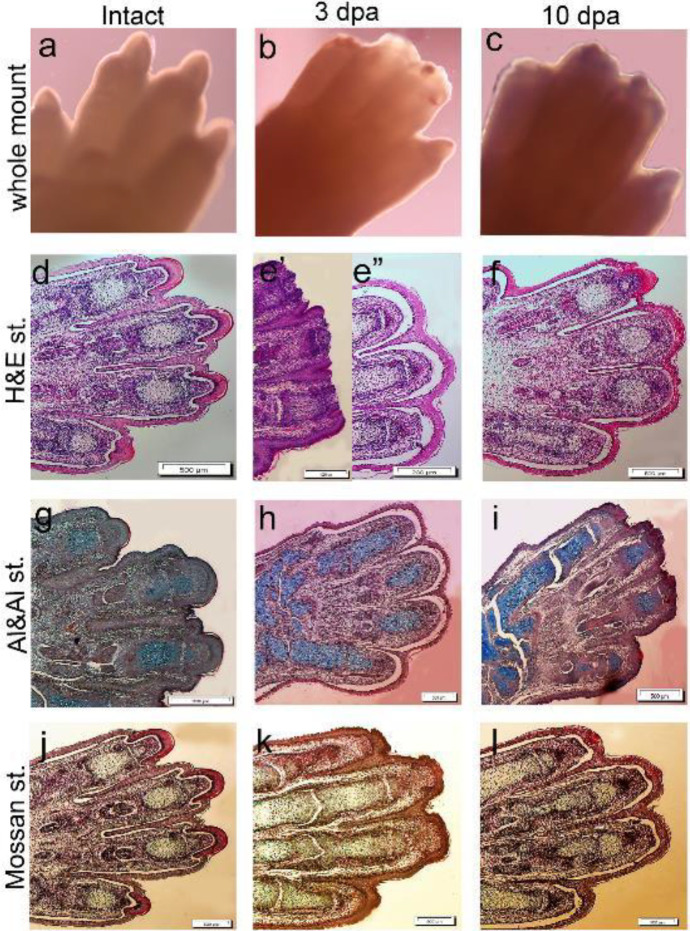
Organ culture system and development of amputated fetal digit tip model. The images show the macroscopic evaluation of whole-mount digit tip of: intact (a), 3 days' post-amputation (dpa) (b), and 10 dpa (c) groups. Histological staining shows epidermal and mesodermal tissue formation in digit tip in the control group (d) and 3 dpa compared with immediate digit tip amputation (e’, e’’) and 10 dpa (f) group. The skeletal elements of digit tip formation by Alizarin red S and Alcian blue staining (g-i). Masson trichrome-stained images confirmed epidermal and mesodermal (bone and cartilage) tissues in the digit tip regrowth (J-I). Scale bar: 200 μm

We developed an *ex vivo* co-culture model of M2c macrophages seeded on a culture plate. The amputated 18–18.5E day mouse forelimb digit tip was placed on a filter insert to assess the cytokine effect of the M2c macrophages on digit tip regeneration at 3 and 10 dpa. The control groups cultured in the same condition media without any M2c cells were used to verify model regeneration prior to co-culture with the M2c cells (co-mac) and treatment with 40, 80, and 120 ng/ml of IL-10 and TGF-β1 cytokines. The whole organ culture showed a scar-free wound closure that formed 2–3 days post-amputation (Figures 3a-c). H&E analysis showed an epidermal closure in our model; the thickness of epidermis and dermis layers had no significant differences between the intact samples and control groups after 3 and 10 dpa. We observed complete closure in the epidermis without any remarkable new tissue formation (cartilage) in our model (Figures 3d-f). However, in our model we detected no apoptotic or necrotic cells. Staining with Alizarin red S (bones), Alcian blue (cartilage), and Masson’s trichrome showed that connective tissue such as cartilage, and dermis and epidermal tissue were fully recognizable in the organ culture model. The cartilage tissue was preserved during the culture time (Figures 3g-l).

***Macroscopic evaluation of digit tip regrowth***

**Figure 4:**
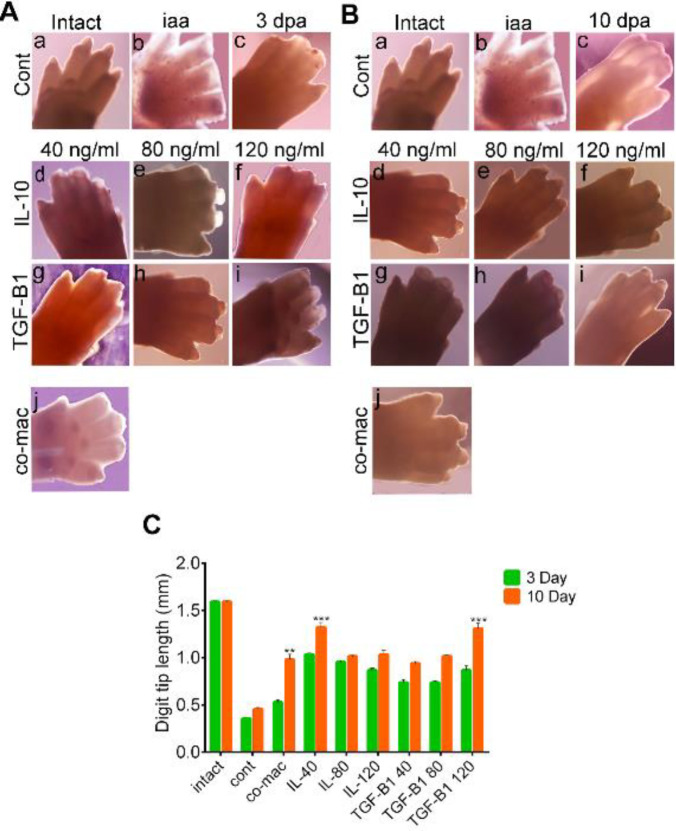
Whole-mount evaluation of digit tips and measurement of elongation. A. Left panel shows whole mount forelimb 3 days’ post-amputation (dpa), including the control (a-c), interleukin 10 (IL-10) (d-f), transcription growth factor-beta 1 (TGF-β1) (g-i), and co-culture with M2c cells (co-mac) (j) groups. B. Right panel shows whole mount forelimb after 10 dpa in the control (a-c), IL-10 (d-f), TGF-β1 (g-i), and co-mac (j) groups. C. Histogram shows the amount of digit tip elongation analyzed by Image-J software on whole-mount samples from each group (n=10). Data are means ±SD. ***P<0.001

We observed closure in the epidermis at the digit tip amputation, followed by wound epidermis formation in all of the experimental and control groups at 3 days after cultivation, which was expected. (Figures 4A, B). We measured digit tip elongation, cell proliferation, and novel tissue formation in the treatment and control groups. Additionally, we compared the amount of digit tip regrowth at days 3 and 10 after cultivation. In the co-mac and cytokine groups, regeneration occurred in more than 90% of the digit tips. Rare elongation occurred in 30% of the control group (Figure 4C). Unpredictably, more than 90% of the digit tips (14 out of 15) showed complete digit tip regeneration, which involved both novel tissue formation and elongation of the new digit cap. We observed this finding in the co-mac, 40 ng/ml, and 120 ng/ml concentrations of IL-10 and TGF-β1 (Figures 4Ad-j and 4Bd-j). In contrast, almost 30%–50% of the digit tips regenerated in the other cytokine groups. We evaluated the amount of new digit cap elongation in the experimental and control groups compared with the intact digits. The results showed full digit tip regrowth that was significant in the co-mac and 40 ng/ml and 120 ng/ml IL-10 and TGF-β1 concentrations compared with the intact group (Figure 4C).

***Histological assessment of digit tip regeneration***

**Figure 5:**
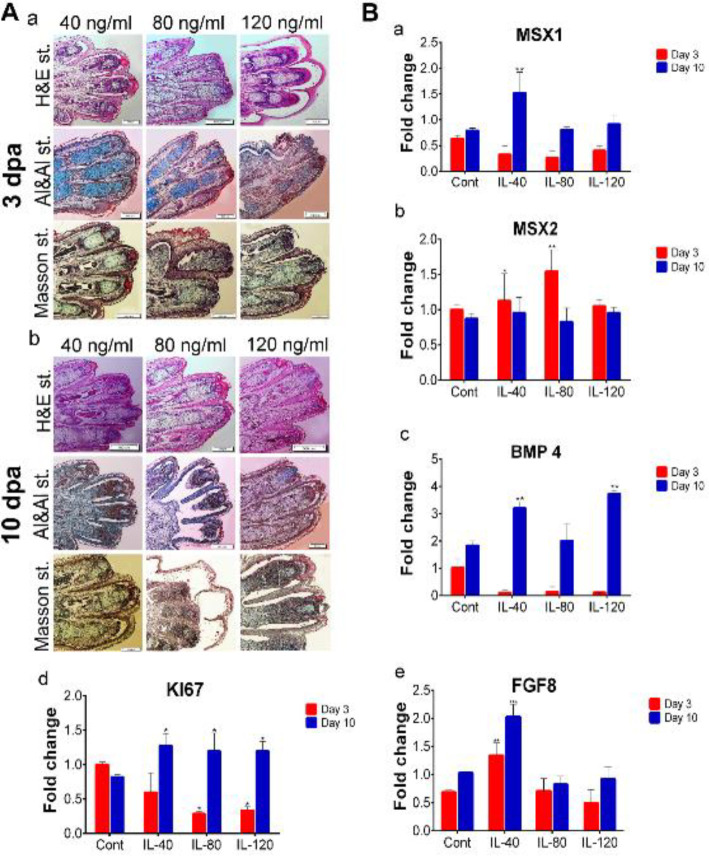
Histological and gene expression analysis of co-culture with M2c macrophage (co-mac) group. A. Hematoxylin and eosin (H&E), Masson's trichrome, Alcian blue and Alizarin red S staining in the M2c and amputated digit co-cultured samples at 3 and 10 days’ post-amputation (dpa). The H&E results showed wound closure at 3 and 10 dpa. No apoptotic cells were found on any of the days. There was new tissue formation on both 3 and 10 dpa. Alizarin red S and Alcian blue images indicated that the region with new tissue stained blue at 10 dpa. Masson’s trichrome staining confirmed new tissue formation at 10 dpa compared with 3 dpa. B. Quantitative reverse transcription-polymerase (qRT-PCR) analyses of Msh homeobox 1 (*Msx1*) expression that significantly up-regulated on 10 dpa (a) and Msh homeobox 2 (*Msx2*) expression showed a non-significant decrease on 10 dpa compared with 3 dpa (b). Additionally, bone morphogenic protein 4 (*Bmp4*) (c), *Ki67* (d), and fibroblast growth factor 8 (*Fgf8*) (e) expressions up-regulated at 10 dpa compared with 3 dpa

**Figure 6:**
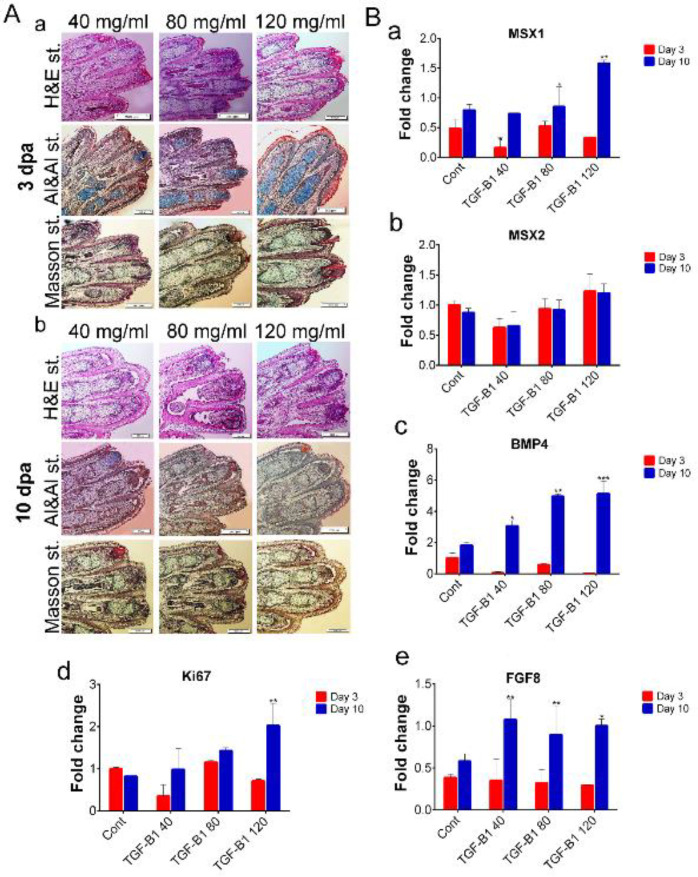
Histological and gene expression assessment of interleukin 10 (IL-10) treatment group. A. Hematoxylin and eosin (H&E) staining illustrates epidermal closure which occurred at 3 and 10 days’ post-amputation (dpa) with all concentrations of IL-10. Alcian blue staining shows cartilage tissue that stained blue in the 40 ng/ml concentration of IL-10 and higher doses of IL-10 showed new tissue formation to bone formation with Alizarin red S staining. A new mass of tissue formed, which stained red with Alizarin red S stain for bone tissue. Masson's trichrome staining confirmed regeneration of the skeletal and connective tissues at all concentrations. B. Quantitative-reverse transcription-polymerase chain reaction (qRT-PCR) data for Msh homeobox 1 (*Msx1*) (a), Msh homeobox 2 (*Msx2*) (b), bone morphogenic protein 4 (*Bmp4*) (c), *Ki67* (d), and fibroblast growth factor 8 (*Fgf8*) (e), as the related regeneration genes after 3 and 10 dpa for 40, 80, and 120 ng/ml concentrations of IL-10 [mean ± SD (n=3)]. **P<0.01

**Figure 7:**
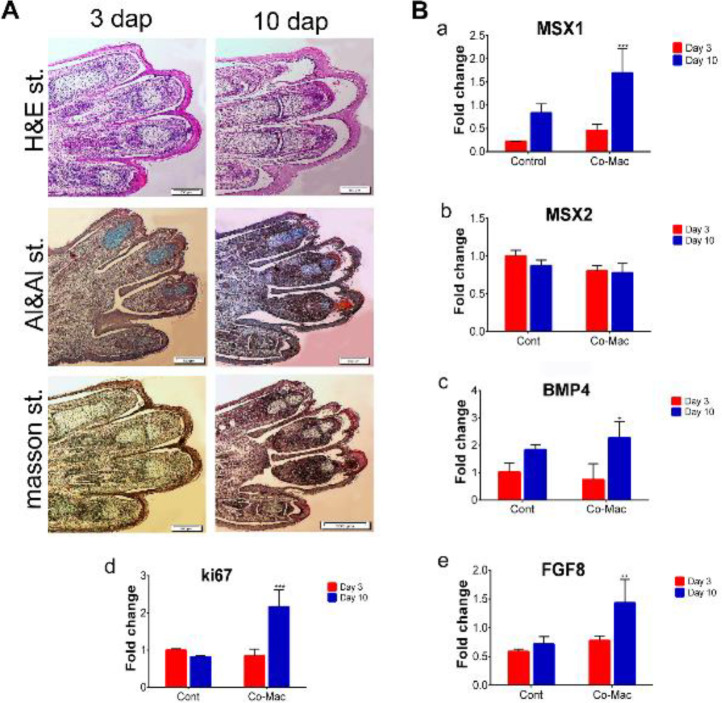
Histological and gene expression evaluation of the transcription growth factor-beta 1 (TGF-β1) treatment group. A. Hematoxylin and eosin (H&E), Masson's trichrome, Alcian blue and Alizarin red S staining in 40, 80, and 120 ng/ml concentrations of transcription growth factor-beta 1 (TGF-β1) at 3 days' post-amputation (dpa) (a) and 10 dpa (b). The H&E results showed wound closure at all concentrations. There were no apoptotic cells in any of the groups. We observed that rare, new tissues formed in all groups. Alizarin red S and Alcian blue images indicated that the region, which contained new tissue was a More intense blue color at the 120 ng/ml concentration compared with 10 dpa. We assumed the presence of skeletal tissues stained by Alcian blue at the 120 ng/ml concentration compared with the lower concentration of TGF-β1. Masson’s trichrome staining confirmed bone formation at the 40 and 80 ng/ml concentrations of TGF-β1 compared with the 120 ng/ml concentration. B. Quantitative reverse transcription-polymerase (qRT-PCR) analyses of Msh homeobox 1 (*Msx1*) expression, which showed significant up-regulation at 10 dpa (a). Msh homeobox 2 (*Msx2*) expression showed no significant up-regulation at 3 dpa compared with 10 dpa at all concentrations (b). Bone morphogenic protein 4 (*Bmp4*) (c), *Ki67* (d), and fibroblast growth factor 8 (*Fgf8*) (e) significantly increased at 10 dpa compared with 3 dpa

 We used H&E staining to determine the presence of apoptotic cells. The results showed the presence of normal skeletal and connective tissue such as epidermis or dermis tissues in our samples. H&E staining results showed that no tissue atrophy and apoptotic cells were present in the connective tissue of the digit stumps in the experiment and control groups at 3 and 10 dpa (Figures 5A, 6A, and 7A). In addition, histological analysis revealed that epidermal closure occurred in all experimental and control groups. The thickness of the dermis and epidermis layers had no significant differences among the experimental and control groups. We performed Alizarin red S (bone), Alcian blue (cartilage), and Masson trichrome staining to visualize the skeletal and connective tissues, and to measure the amount of new tissue formation of each digit cap (20 digits for each group). As shown in Figures 5A, 6A, and 7A, we observed the cartilage stump blue-colored in the co-mac group. The amount of significant digit elongation was variable in different concentrations of IL-10, TGF-β1, and in the co-mac group at 3 dpa compared with 10 dpa. The cartilaginous mass that resulted from the regrowth was significantly larger in the co-mac, 40, and 120 ng/ml concentrations of IL-10 and TGF-β1 10 dpa than in the control group (Figures 5A, 6A, and 7A). On the other hand, morphological analysis determined that digit tip generated up to 60%, 85%, and 75% of the intact group in the co-mac, 40 ng/ml of IL-10, and 120 ng/ml of TGF-β1 groups, respectively (Figure 4C). This tissue mass was replaced by tissue like the cartilaginous segment in the digit tip of fetal mice phalanges. Interestingly, a new digit tip formed, and especially the cartilaginous part typically formed in the co-mac groups, which was significantly like the intact groups (Figure 7A).

***Verification of regeneration-related genes***

 We conducted RT-PCR analysis of several of the regeneration-related genes *Msx1, Msx2, Bmp4, *and *Fgf8 *conserved in mouse and salamander limb regeneration. As shown in Figures 4-6B, the *Msx1* gene had significantly greater expression at 40 and 120 ng/ml concentrations of IL-10 and TGF-β1 compared with the control group. In the co-mac group, *Msx1* expression had a significant 4-fold increase compared with the control group on 10 dpa (**P<0.001, *P<0.01; Figures 5-7Ba). Our analysis showed that the expression level of *Msx2* either decreased or maintained expression up to 10 dpa compared with 3 dpa in all groups, but it did not lead to a significant reduction in the experimental groups compared with the control group (**P<0.001, *P<0.01; Figures 5-7Bb). RT-PCR results showed approximately 4-fold *Bmp4* expression in 40 ng/ml IL-10; 6-fold expression in 120 ng/ml TGF-β1; and 3-fold expression in the co-mac group compared with the control group, which was expected (**P<0.001, *P<0.01; Figures 5-6Bc). *Fgf8* expression significantly up-regulated in the 40 ng/ml concentration of IL-10 (**P<0.01; *P<0.01; Figure 6Be). Expression levels of *Fgf8* increased significantly at each concentration of TGF-β1 compared with the control (**P<0.01; *P<0.01; Figure 7Be). The expression level of *Ki67* was significantly elevated in all experimental and control groups at 10 dpa compared with 3 dpa. Increased *Ki67* expression levels were observed in all concentrations of IL-10 at 10 dpa compared with 3 dpa. In TGF-β1, high-levels of *Ki76* expression persisted in all concentrations, but it was significantly greater at 120 ng/ml than at 40 and 80 ng/ml concentrations (*P<0.01; Figures 5-7Bd).

***IHC and protein expression analysis***

**Figure 8:**
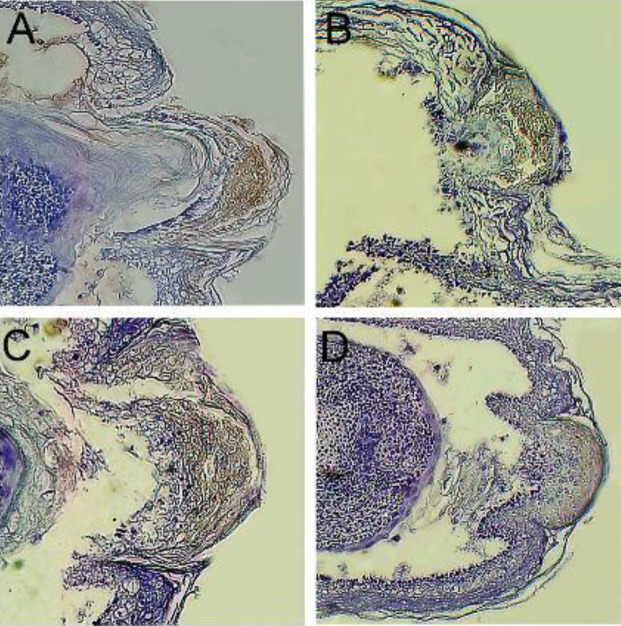
Immunohistochemistry (IHC) analysis of Msh homeobox 1 (MSX1), Msh homeobox 2 (MSX2), bone morphogenic protein 4 (BMP4), and fibroblast growth factor 8 (FGF8). Immunohistochemistry (IHC) staining showed that Msh homeobox 1 (MSX1), Msh homeobox 2 (MSX2), bone morphogenic protein 4 (BMP4), and fibroblast growth factor 8 (FGF8) were expressed in the co-culture with M2c cells (co-mac) group. A. MSX1, B. MSX2, C. BMP4, and D. FGF8

 We performed IHC staining for the presence and location of MSX1, MSX2, FGF8, and BMP4 proteins in the tissue sections. In the co-mac group, the protein expressions of MSX1, MSX2, FGF8, and BMP4 dramatically increased in the regrown digit cap (Figures 8A-D). These results supported the RT-PCR results of the expression profiles of *Msx1*, *Msx2*, *Fgf8*, and *Bmp4*. Likewise, IHC analysis indicated that *MSX1* and *MSX2* genes up-regulated in newly formed epidermal and mesodermal tissues, which are the main markers of ecto-mesodermal non-differentiated tissue in limb development (Figures 8A, B). We observed BMP4 expression was up-regulated following an increase in the expression of the MSX1 gene, which was expected (Figures 8A, B, and D). 

## Discussion

Limb or digit tip regeneration may fail in adult mammals as the result of pro-inflammatory responses in the presence of scar formation (45, 46). Neutrophils and macrophages are crucial mediators of the anti-inflammatory process, whose main roles have been demonstrated in regrowth of the limbs, heart, and tail in salamanders. However, they have not been extensively studied in adult mammals (17). On the other hand, in adult salamanders, simultaneous induction of pro- and anti-inflammatory signals in the amputated limb area result in scar-free full regeneration of the limb (15). Here we have examined the effect of the anti-inflammatory M2c macrophages on regrowth of mice forelimb digit tips immediately after amputation. We utilized a co-cultured system of macrophages combined with the forelimbs of mice that had three digit tips amputated and three concentrations, separately, of TFG-β1 and IL-10. Peripheral blood derived-monocyte cells were used for M2C macrophage production. Previous studies showed that more than 95% of peripheral blood cells are erythrocytes and 5% are other cells such as thrombocyte, granulocyte, lymphocyte, and monocyte, and that monocytes are adherent cells and other cells are floating cells (42). So, the monocyte cells were detached from floating cells following their adherent ability to the bottom of the dish. Isolated cells converted to macrophages using IL-10. Then, we measured their effects on mouse digit tip regeneration. Our data have supported the hypothesis that the presence of anti-inflammatory responses at the site of the injury immediately after amputation is a crucial step for mice digit tip regeneration, which is similar to adult salamanders, where macrophages and neutrophils migrate to the injured site via circulation. Whereas, partial macrophage depletion in limb tissues and peripheral blood before the amputation caused widespread prevention of salamander’s limb regeneration. In addition, permanent scar tissue formed in the absence of macrophages in the stumps of the amputated limbs in salamanders (15). 

Recently, it has been reported that similar macrophages exist in the heart, which migrate to the sites of heart injury in neonatal mice. Furthermore, these cells are functionally important for heart wound healing and zebrafish tail regeneration (36). Alternatively, immediate macrophage depletion did not cause failure of epithelial wound closure after limb amputation in the axolotl; instead, it caused excessive fibroplasia or collagen deposition followed by fibrotic formation (15). According to the literature, early pro- or anti-inflammatory response are key determinants of whether there is scar formation or regeneration in mammalian organs at 48–96 hr after an injury. Macrophages that are present at the regenerating axolotl blastemal site 24 hr after amputation, followed by simultaneous induction of inflammatory and anti-inflammatory cytokines are the main reason for full limb regeneration. We designed a co-cultured system that mimicked the salamander’s anti-inflammatory responses in the amputated digit tip of fetal mice. First, we derived mononuclear cells and monocyte precursors from peripheral blood. Previous studies demonstrated that monocytes respond to environmental cues within tissues such as damaged cells, activated lymphocytes, or microbial products to differentiate into distinct functional phenotypes in the *in vivo *environment. The IL-10 cytokine has been used to push prior monocytes toward M2c macrophages as the anti-inflammatory subtype. Analysis of morphological and phagocytosis activity showed that monocytes convert to a macrophage-like phenotype (Figures 2Aa-c). ELISA and ICC analyses confirmed that macrophages accurately converted to the M2c subtype immediately after IL-10 stimuli and before they entered apoptosis (Figures 2B, Ca, b). High-level expressions of surface biomarkers such as CD68, CD80, and MHC II are the main M1 macrophage phenotypes. In contrast, M2 macrophages are determined by their association in parasite control, tissue remodeling, immune regulation, tumor promotion, and efficient phagocytic activity (47). M2c macrophages are a subtype of M2 macrophages characterized by IL-10, TGF-β, and glucocorticoids and they lack CD68, MHC II markers as M1 marker expression (48). 

Our ELISA analysis revealed that the maximum amount of TGF-β1 cytokine as the main M2c secretion was released three days after IL-10 stimuli prior to reduction as a result of macrophage apoptosis. So, we considered the amount of regeneration during 10 dpa. We explored the regenerative ability of an amputated forelimb digit tip after co-culture with M2c macrophages and either 40, 80, or 120 ng/ml dosages of IL-10 and TGF-β1 in a fetal mice model. In our model, we preserved the cartilaginous element of the joint from the third phalange and detached the cartilage, dermal, and epidermal tissues of the digit tips. Whole-mount evaluation showed the most digit tip regrowth occurred in the co-mac groups, and the 40 and 120 of dosages of IL-10 and TGF-β1 cytokines on 10 dpa (Figures 4A, B). More importantly, histomorphometric measurement confirmed the significant digit tip elongation in the co-mac, 40 ng/ml, and 120 ng/ml concentrations of IL-10 and TGF-β1, where there was complete new digit tip formation, which was like the intact group after 10 dpa. We assessed the histological and expression levels of regeneration-specific markers *Msx1*, *Msx2*, *Bmp4,* and *Fgf8* in all groups to address the recent tissue formation observed in the experimental groups. 

H&E staining showed that apoptotic or dead cells were not identified in the experimental and control groups up to 10 dpa. In addition, epithelial wound closure occurred in both the control and experimental groups at 3 dpa, which was expected (Figures 4A, 5A, 6A). Masson’s trichrome staining was used to determine the tissue type formed in the new callus regrowth (Figures 5A, 6A, 7A). Epidermis tissue stained an amethyst color that accurately confirmed the epidermal closure occurred in all groups. The connective tissue stained red and the digit stump showed slight elongation in all experimental groups. The identity of callus was significantly not clear in any of the groups. In order to precisely address which, type of skeletal tissue formed, we performed Alizarin red S and Alcian blue staining. According to the results, the co-cultured system induced new cell proliferation in the digit tips. Alizarin red S and Alcian blue staining showed new callus formation that stained blue and a rare bony-like tissue formation that stained red by Alizarin red S at 10 dpa in the co-mac group. Interestingly, in the IL-10 and TGF-β1 groups, new regrowth tissue was observed with 40 and 120 ng/ml concentrations, respectively. Although digit tip elongation significantly increased in the co-mac, 40 and 120 ng/ml concentrations, from the point of histology there were no remarkable differences between the three groups. Real-time PCR results showed that the highest expression levels of *Msx1, Bmp4, Ki67, *and* Fgf8* genes were observed in the 40 and 120 ng/ml concentrations at 10 dpa. As expected, the expression level of *Msx2* did not have significant up-regulation in 10 dpa compared with 3 dpa.

Interaction of AER as a thickening of the epidermal cells and underling mesenchyme has been demonstrated to support limb outcome (49). The homeobox gene, *Msx1*, is related to the distal limb mesenchyme and its expression depends upon the existence of AER in limbs as well as *Msx2* gene expression (50). When the digit tip is removed, or when the amputated forelimb digit tip is placed in a co-culture system, there are detectable expression levels of *Msx1*, *Msx2*, and *Bmp4* in the mesenchymal region of the digit (8). Our IHC results demonstrated that the expression of *Msx1* up-regulated in the distal mesenchyme as well as the digit epidermal layer in the newly formed digit tips after 10 dpa. *Msx1* can also up-regulate *Bmp4* gene expression as a skeletal marker in the mesenchyme region, which was confirmed by real-time PCR assessment. However, *Msx2* expression was not significantly up-regulated on 10 dpa compared with 3 dpa. IHC analysis confirmed the Wound closure that occurs during the first three days after an amputation may, after a brief period, expand into the mesoderm along the distal mesenchyme of the growth area. The proximo-distal patterning of limb/digits tissue is negatively controlled by the Msx2 gene, which regulates the outgrowth of the limb mesenchyme region and associated structures to the distal mesenchyme (51). In our previous study, we showed that blastemal-like cells produced with mesenchymal stem cells (MSCs) overexpressed by *Msx1* and *Msx2* genes had full regenerative potential of the amputated digit tip in adult mice. Our findings allowed us to introduce alternative cell sources that provide positional information similar to that of blastema cells. Previous studies showed MSCs have no ability for digit tip regeneration or digit patterning (8). The outgrowth and polarizing area of the AER are controlled by subfamily members of fibroblast growth factors (FGFs) as well as *Fgf8* in limb development(52). Here, we have shown *Fgf8* expression in the epidermal border in the ridge of the mesenchyme.* Fgf8* expression was observed in the mesenchyme following ridge removal. We sought to explain the initial anti-inflammatory response required for mouse digit tip regeneration, which occurs during limb regeneration of adult salamanders. Our results verified that M2c macrophages co-cultured with amputated forelimb digit tips fully regenerated proximally amputated digit tips in fetal mice. This study showed that the anti-inflammatory cytokines, TGF-β1 and IL-10, exhibited similar effects to M2c and up-regulation of *Msx1 *and *Msx2*, as well as *Bmp4* and *Fgf8, *compared with IL-10 and TGF-β1. This finding has proposed that the increased anti-inflammatory cytokines, which contain essential genes that activate endogenous signaling pathways, effectively accelerate the regeneration process. Further experiments are necessary to clearly elucidate the effect of M2c as an anti-inflammatory macrophage on amputated adult mouse digit tips *in vivo*. 

M2c-macrophages up-regulated regeneration-associated genes MSXs, FGF8 and BMP4 resulting in epidermal closure and new tissue regrowth at the digit tip wound site. IL-10 and TGF-β mimicked the regenerative effects of M2c-macrophages in a dose-dependent manner on the mice amputated paw

**Table 1:** Description of mouse primers used in quantitative reverse transcription-polymerase chain reactions (qRT-PCR)

Gene symbol	Sequences(5-3)	Accession no.	Amplicon size (bp)
**Gapdh** **Fgf8** **Bmp4** **MSX1** **MSX2** **Ki67**	FOR: 5' ACTTCAACAGCAACTCCCAC3' REV: 5' TCCACCACCCTGTTGCTGTA3' FOR: 5' GGGGAAGCTAATTGCCAAGA3' REV: 5' CCTTGCGGGTAAAGGCCAT 3' FOR: 5' GTCGTTTTATTATGCCAAGTC3' REV: 5' ATGCTGCTGAGGTTGAAGAG3' FOR: 5' CTGCTATGACTTCTTTGCC 3' REV: 5' CTTCCTGTGATCGGCCAT 3' FOR: 5' CACCACATCCCAGCTTCTA 3' REV: 5' GCAGTCTTTTCGCCTTAGC 3' FOR: 5' CTGCCTCAGATGGCTCAAAGA 3' REV: 5' GAAGACTTCGGTTCCCTGTAAC 3'	NM_008084 NM_001166361. NM_001316360. NM_010835.2 NM_013601.2 NM_001145966.	60⁰C 60⁰C 60⁰C 60⁰C 60⁰C 60⁰C

## Conclusion

The present experiment demonstrated the role of M2c macrophages on mice digit tip regeneration *in vitro*. M2c macrophages can induce regeneration-associated genes MSX1, MSX2, FGF8 and BMP4 at the wound site. Epidermal closure and new tissue regrowth occur in amputated digit tip during co-culture with M2c macrophages. In addition, IL-10 and TGF-β mimic the regenerative effects of M2c-macrophages in dose depended manner on the mice amputated paw.

## Authors’ Contributions

FB performed the surgery and behavioral and histological experiments; LT performed image analysis and wrote the manuscript; ZA performed immunohistochemical studies; and M B E conceptualized the study and wrote the manuscript. All authors contributed to data analysis, drafting and revising the article, gave final approval of the version to be published, and agreed to be accountable for all aspects of the work.

## Statement of Ethics

All procedures were approved by the Animal Ethics Committee at the animal department of Royan Institute and were according to the international regulations of the European Directive 2010/63/EU and ethical guidelines for the study of experimental pain in conscious animals by the International Association of the Study of Pain.

## Conflicts of Interest

The authors declare no conflicts of interest.
